# Supplementary material for: Enhanced pupillary light reflex in infancy is associated with autism diagnosis in toddlerhood
Source: Nat Commun. 2018 May 7;9:1678. doi: 10.1038/s41467-018-03985-4 (PMC5938234; doi:10.1038/s41467-018-03985-4)
Supplement: Supplementary file 1 — Supplementary Information [file 41467_2018_3985_MOESM1_ESM.pdf]

SUPPLEMENTARY INFORMATION

Enhanced pupillary light reflex in infancy is associated with autism diagnosis in toddlerhood

Nyström, et al.

Supplementary Figures

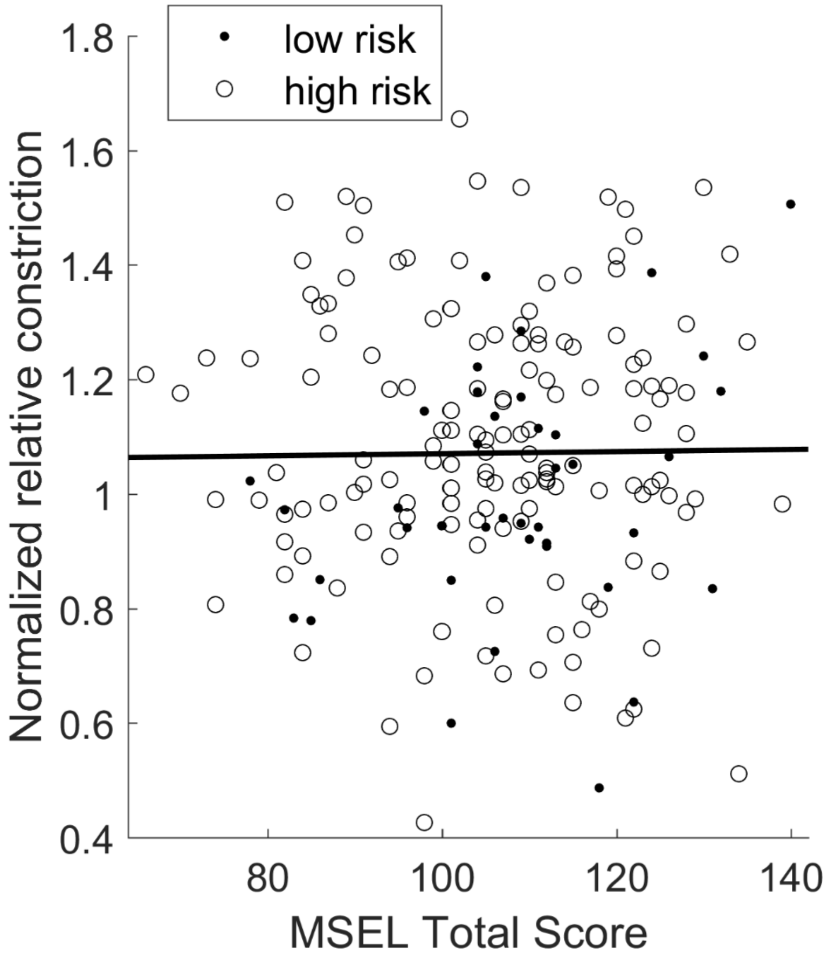

Supplementary Fig. 1. *Scatterplot of normalized relative constriction versus Mullen Scales of Early Learning (MSEL) total score at 9-10 months of age.*

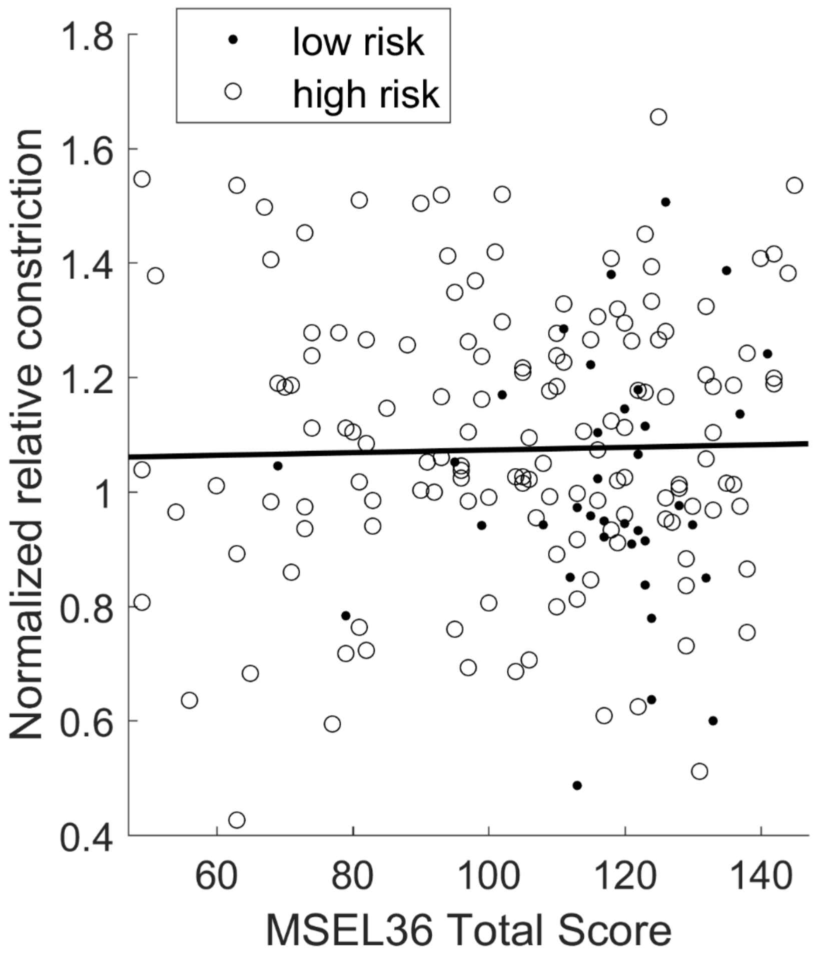

Supplementary Fig. 2. *Scatterplot of normalized relative constriction in infancy versus Mullen Scales of Early Learning (MSEL) total score at 36months of age.*

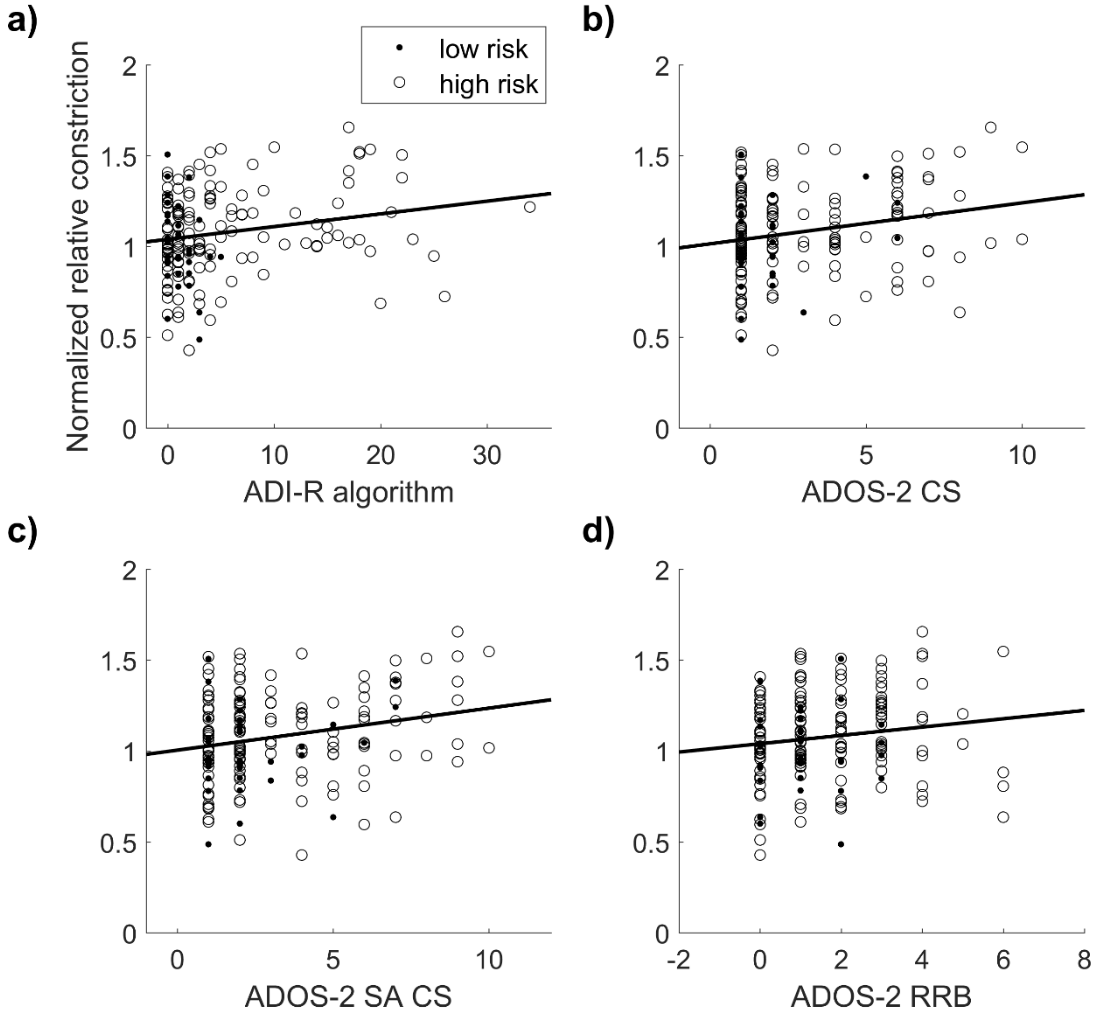

Supplementary Fig. 3. *Scatterplots showing the association between normalized relative constriction of the PLR in infancy and severity measures of ASD symptoms at three years of age, including all participants. Large relative constriction of the PLR in infancy was associated with having higher* ***(a)*** *ADI-R scores,* ***(b)*** *ADOS-2 comparison scores, and* ***(c)*** *Social Affect (SA CS) comparison scores (ADOS-2 subscale). There was also a trend for the Restricted and Repetitive Behaviors (RRB) algorithm scores (ADOS-2 subscale;* ***(d)****). Statistics in main text.*

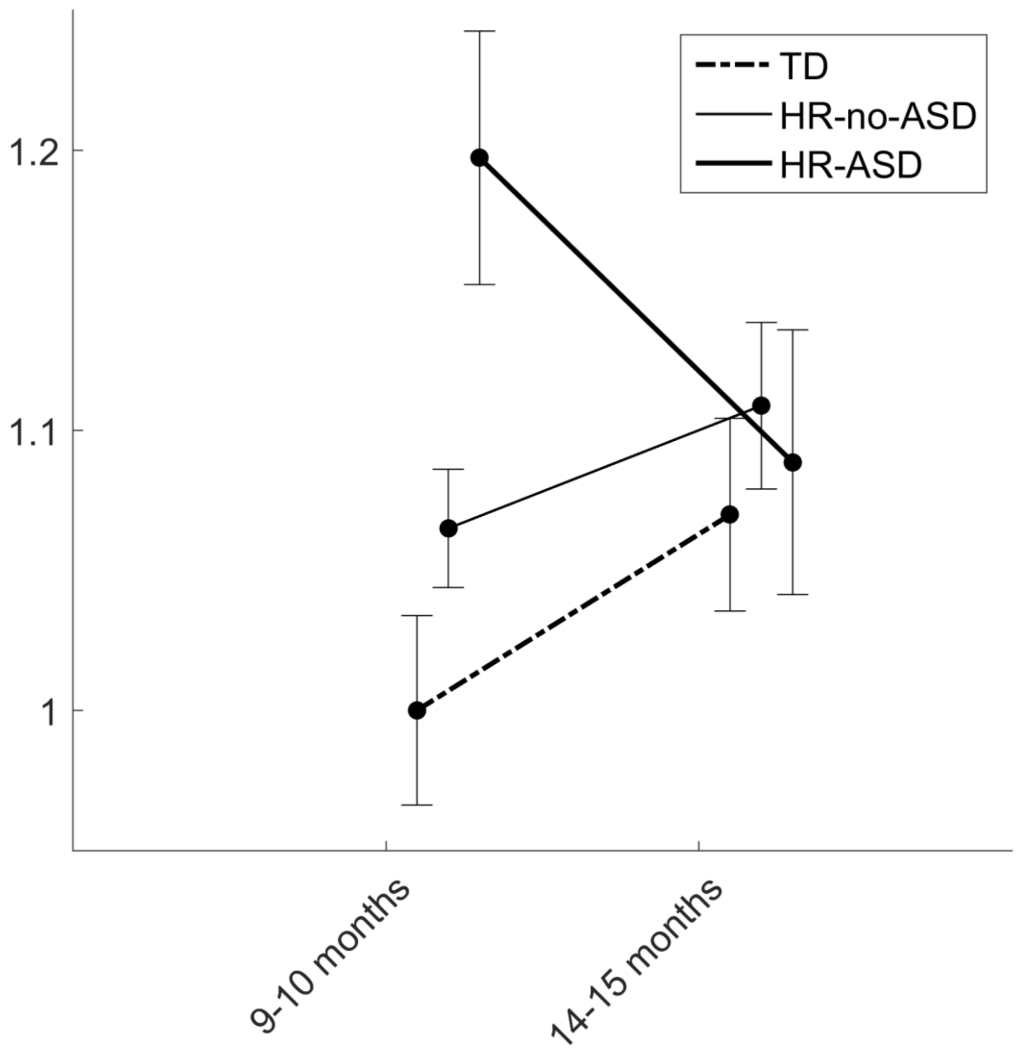

Supplementary Fig. 4. *Data of relative constriction from both time points. As in the main analysis, all means are normalized according to the TD group's mean at 9-10* months. *Error bars show s.e.m..*

Supplementary Methods

1. Supplementary participant information

1.a. EASE sample

ASD diagnosis of the older sibling (hereafter: proband) in the HR group was confirmed by a psychologist led interview with parents and by reviewing medical records (in more than 70% of cases it was explicitly stated that the Autism Diagnostic Observation Schedule (ADOS) and/or the Autism Diagnostic Interview-Revised (ADI-R) were used as part of the diagnostic evaluation). Infants in the low-risk control group were recruited from a volunteer database. Inclusion criteria included full-term birth and lack of any ASD within second-degree family members (as confirmed through parent interview regarding family medical history). All included low risk infants had at least one older sibling. All families received a compensation of € ~50 for their participation. The final EASE sample consisted of 15 infants in the TD group, 13 in the HR-ASD group, and 25 in the HR-no-ASD group.

1.b. BASIS sample

High-risk infants had at least one older sibling with a community clinical diagnosis of ASD. Proband diagnosis was confirmed by an expert clinician (TC) based on information using the Development and Well-Being Assessment (DAWBA)[1] and the parent-report Social Communication Questionnaire (SCQ)[2]. Parent-reported family medical histories were examined for significant medical conditions in the proband or extended family members, with no exclusions made on this basis. Infants in the low-risk control group were recruited from a volunteer database. Inclusion criteria included full-term birth, normal birth weight, and lack of any ASD within first-degree family members (as confirmed through parent interview regarding family medical history). All low-risk participants had at least one older sibling.

Screening for possible ASD in these older siblings was undertaken using the SCQ, with no child scoring above instrument cut-off for ASD. The final BASIS sample consisted of 25 infants in the TD group, 16 in the HR-ASD group, and 93 in the HR-no-ASD group.

2. Extraction of the pupillary light reflex parameters

Pupil samples outside the range 1-10 mm diameter were considered artefactual and removed. Short gaps in the data series (<7 samples) with <0.2 mm pupil change were linearly interpolated, to account for occasional "flicker" in eye tracking data collection and create continuous pupil traces for artefact rejection. All samples with a change in pupil size of >0.3 mm/sample were removed, and all data segments shorter than 5 samples were removed, as such data islands typically deviated from the surrounding pupil readings. An additional linear interpolation of missing data (same settings as above) was performed. To achieve better temporal resolution[3] the data was resampled to 300 Hz. First and second order derivatives of the pupil size, yielding pupil velocity and acceleration respectively, were calculated using the MATLAB diff function, and 25-point moving average filters were applied to the pupil size, pupil velocity and pupil acceleration to reduce amplification of noise during derivation[4].

The PLR latency was defined by the acceleration minima in the time interval 100 - 400 ms relative stimulus onset[3,4]. The baseline pupil size for each trial was defined as the average pupil size in a 100 ms interval just before the latency time point. The relative constriction of the PLR was calculated as described in the main text, within the interval 500– 1500 ms after the flash.

Trials were excluded if 1) the latency interval (100 - 400 ms relative stimulus onset) had more than 75% interpolated data; 2) if the relative constriction interval (500–1500 ms) had more than 75% interpolated data; 3) if the relative constriction was outside a range of 5 to 80 percent, out of which the PLRs were too small to be visually distinguished from noise, or to

high to be biologically plausible); 4) if the latency or max relative constriction time points were in or adjacent to interpolated data. This automatic exclusion procedure was validated by visual inspection blinded to outcome group (pupil traces from left and right eye for each trial was inspected separately), and trials that did not resemble a PLR were manually rejected. Only one eye contributed with PLR data; data from the left or right eye that correlated best with the individual's mean trace (i.e. all valid trials averaged within subject) was selected automatically.

Infants with less than three valid trials at the 9-10 month time point were excluded from further analysis (total *n*=14; TD *n*=4; HR-no-ASD *n*=5; HR-ASD *n*=5). Q-Q plots were used to inspect data distributions for outliers and deviations from normality before statistical analysis and to inspect residuals after model fitting.

Supplementary note 1

1.a. MSEL

To investigate whether the main finding could be confounded with a general intelligence factor we included the MSEL as a covariate in the main model (i.e. relative constriction at 9-10 months as dependent variable, group as fixed factor, and MSEL at 9-10 months as covariate. The results showed no effect of the MSEL: $F(1, 183)=0.024$, $P=0.495$, $\eta p^2=0.003$, and the other effects remained unchanged. Although the MSEL did not distinguish between groups, the scores might share some meaningful relationship with the PLR. To assess this at the most general level we performed Pearson correlations of all participants pooled together ($n=187$). The results did not show any significant association between MSEL and the relative constriction: $r=0.012$, $P=0.872$ (see **Supplementary Fig. 1**). Similarly, we found no relationship between relative constriction (9-10 months) and MSEL at 36 months (**Supplementary Fig. 2**).

1.b. Missing data

Another possible confound is that the data quality from participants varied in systematic ways between groups. To investigate this issue, we calculated the amount of missing data for each trial and used the average within participants as a dependent variable in a GLM with group as fixed factor. Because the dependent variable did not show equal error variances between groups (Levene's test) we ran the analysis again on the log transformed dependent variable. This model did not show any significant differences between groups: $F(2, 191) = 0.360$, $P=0.698$, $\eta p^2=0.004$, and neither did any pairwise comparison between groups. Further, adding the amount of missing data as a covariate in the main relative constriction analyses did not show any interaction effect with group: $F(2, 181)=0.088$, $P=0.916$, $\eta p^2=0.001$, and the main effect of group and pairwise comparisons between groups remained unchanged. This suggests that differences in data quality between groups did not confound our main findings.

1.c. Gender differences

To investigate any possible influence of gender on the main results we performed a GLM with relative constriction as dependent variable, and group and gender as fixed factors. The results showed no main effect of gender: $F(1, 181)=1.130$, $P=0.289$, $\eta p^2=0.006$), no interaction effect between gender and group: $F(2, 181)=0.101$, $P=0.904$, $\eta p^2=0.001$, while the main effect of group $F(2, 181)=4.160$, $P=0.017$, $\eta p^2=0.044$, and the pairwise comparisons between groups, did not change significance. Further, when adding gender as a fixed factor in the dimensional analyses (now running GLMs instead of pearson correlations) did not show any significant main effects of gender or interaction effects with the dimensional measure, while all dimensional factors (ADI-R Total, ADOS CSS, ADOS SA, and ADOS RRB) remained

significant. Thus, there are no apparent gender differences in terms of the PLR response in this context.

1.d. Sensitivity analysis (reference group)

As noted in the main text, we used the TD group as our first choice for site normalization, as we assumed that this group was most likely to be similar across our two sites given the similar context (recruited from volunteer database for "babylabs", both being situated in major cities in Europe). However, as the group comparison for the combined dataset is influenced by this choice, we repeated the main analysis using the other groups as normalization as well. With the HR-ASD group as normalization group, we observed the following pattern: HR-ASD vs TD, P=0.001, HR-ASD vs HR-no-ASD, P=0.036, HR-no-ASD vs TD, P=0.049 (two tailed probabilities, uncorrected). With the HR- no ASD group as normalization group, we observed the following pattern: HR-ASD vs TD, P=0.002, HR-ASD vs HR-no-ASD, P=0.060, HR-no-ASD vs TD P=0.039. Thus, overall, the pattern reported in the main text was replicated, a result that should be interpreted in light of our directional hypothesis[3].

Supplementary note 2

2. Data at 9-10 months

2.a. PLR latency

The same GLM model structure as in the main text was used to assess differences between groups, with group and fixed factor and TD normalized latency as dependent variable. As noted in the main text there was no significant main effect of group, and descriptive statistics were as follows: TD, n=40, mean=1.000, SD = 0.109; HR-no-ASD, n=118, mean=1.048, SD = 0.138; HR-ASD, n=29, mean=1.014, SD = 0.140. Planned comparisons between groups did not show any significant differences: HR-ASD vs TD, P=0.666, 95% CI [-0.050 to 0.078]);

HR-ASD vs HR-no-ASD, *P*=0.220, 95% CI [-0.088 to 0.020]; HR-no-ASD vs TD, *P*=0.0503, 95% CI [-0.000 to 0.096], although the last comparison showed a trend of elevated latencies in the HR-no-ASD group.

2.b. Baseline pupil size

Because there could be group differences in arousal that affect the pupil responses we tested whether the baseline pupil size differed between the groups. In this analysis the TD normalized baseline was used as the dependent variable and group was used as fixed factor. There was no significant main effect of group: $F(2, 184)=1.454$, $P=0.236$, $\eta p^2=0.016$, and descriptive statistics were TD, *n*=40, mean=1.000, SD = 0.099; HR-no-ASD, *n*=118, mean=1.039, SD = 0.130; HR-ASD, *n*=29, mean=1.030, SD = 0.130. Planned comparisons between pair of groups showed that none of the groups differed in terms of their pupil size at baseline: HR-ASD vs TD, *P*=0.325, 95%CI [-0.030 to 0.090]; HR-ASD vs HR-no-ASD *P*=0.731, 95%CI [-0.060 to 0.042]; HR-no-ASD vs TD *P*=0.090, 95%CI [-0.006 to 0.084]. Also, adding the TD normalized baseline values as a covariate in the main analysis of relative constriction did not change the main effect of group or any of the pairwise comparisons, which suggests that our main finding was not biased by baseline values.

Supplementary note 3

3. Site effects 9-10 month

The two sites presented different types of stimuli to elicit PLR responses, and we accounted for site related variability by normalizing all values by each site's average TD value. To investigate group differences within each site, we performed the same GLM as in the main analysis for each site separately. In sum, these analyses show that descriptively, the same pattern of group differences (TD < HR-no-ASD < HR-ASD) replicates in both sites, but that

the pattern of significant results in specific tests differ between sites. In the following analyses the GLM models used TD normalized relative constriction as dependent variable and group as fixed factor.

3.a. EASE relative constriction

The results showed a significant main effect of group, $F(2, 50) = 11.958$, $P<0.001$, $\eta p^2=0.324$, and planned comparisons showed a difference between the HR-ASD and the TD group ($P<0.001$, 95% CI [0.157 to 0.402]) and between the HR-no-ASD and the TD group ($P<0.001$, 95%CI [0.100 to 0.312]), but not between the HR-ASD and the HR-no-ASD group ($P=0.188$, 95%CI [-0.037 to 0.184]). Descriptive statistics for the groups were TD $n=15$, mean=1.000, SD = 0.184; HR-no-ASD $n=25$, mean=1.206, SD = 0.144; HR-ASD $n=13$, mean=1.279, SD = 0.166.

3.b. BASIS relative constriction

In the BASIS cohort the results did not show a significant main effect of group: $F(2, 131) = 1.585$, $P=0.209$, $\eta p^2=0.024$). Planned comparisons showed a marginally significant difference between the HR-ASD and the TD group ($P=0.092$, 95% CI [-0.021 to 0.282]), but not between the HR-no-ASD and the TD group ($P=0.617$, 95%CI [-0.080 to 0.134]), or between the HR-ASD and the HR-no-ASD group ($P=0.113$, 95%CI [-0.025 to 0.231]). Descriptive statistics for the groups were TD $n=25$, mean=1.000, SD = 0.234; HR-no-ASD $n=93$, mean=1.027, SD = 0.234; HR-ASD $n=16$, mean=1.130, SD = 0.279.

Because the PLR was measured following a black gap between two white stimuli in the BASIS sample, we investigated whether the gap length, which varied slightly between subjects as stated in the main text, had any impact on the relative constriction results in the BASIS sample. We added the gap length as a covariate to the GLM model and investigated both main and interaction effects with group. The results did not show any significant main

effect of gap length: $F(1, 128)=1.731$, $P=0.191$, $\eta p^2=0.013$, nor a significant interaction: $F(2, 128)=1.531$, $P=0.220$, $\eta p^2=0.023$), suggesting that this possible error source did not influence the other results.

In the BASIS sample, after the first time point 54 of the high risk participants was enrolled in a randomized controlled trial (RCT) of parent-mediated intervention[5] and another four participants in a similar non-RCT intervention. To rule out any confounding contributions from the intervention programmes we performed the following analyses using two binary factors as such: 1) To account for differences due to different sampling of the groups recruited or not recruited for intervention, we tested for a main effect of a recruitment factor (1=participating in the RCT, 0 =not part of the RCT); 2) To account for effects of intervention treatment on the relative constriction, we tested for a main effect of the treatment factor (1=treated, 0=not treated); 3) To account for any moderating effect treatment might have had on the relationship between relative constriction and diagnostic outcome, we tested for an interaction effect between treatment and diagnostic outcome. Because there were no significant effects in any these analyses, neither the recruitment factor nor the treatment factor were used in any further analysis.

Supplementary note 4

4. Longitudinal analysis of relative constriction

As stated in the main text, our finding of increased PLR in HR-ASD at 9-10 months of age are opposite to previous findings of decreased PLR in children with ASD[6]. We therefore asked whether developmental changes occur sometime between infancy and middle childhood which would lead to a crossover effect between HR-ASD and TD groups. Our data allowed for a longitudinal analysis of the relative constriction at 9-10 months and 14-15 months. Despite the short time span we found a significant difference in the change scores between the

HR-ASD group (n=21, mean=-0.057, SD=0.214) and TD group (n=27, mean=0.102, SD=0.248). In other words, the HR-ASD group decrease and the TD group increase in relative constriction between 9-10 and 14-15 months in our sample. Note that the number of participants is slightly lower than in the primary analyses, which is due to dropouts in the longitudinal design (i.e. some participants had less than three valid trials at the 14-15 month time point). The relative constriction data from all groups and time points were as follows were as follows (see also **Supplementary Fig. 2**): TD, 10m: n=40, mean=1.00, SD = 0.21, 95% CI [0.966 to 1.034]; 14m: n=31, mean=1.07, SD = 0.19, 95% CI [1.035 to 1.104]; HR-ASD, 10m: n=29, mean=1.20, SD = 0.24, 95% CI [1.152 to 1.242]; 14m: n=26, mean=1.09, SD = 0.24, 95% CI [1.041 to 1.136].

Supplementary References

1 Goodman, R., Ford, T., Richards, H., Gatward, R. & Meltzer, H. The Development and Well-Being Assessment: description and initial validation of an integrated assessment of child and adolescent psychopathology. *The Journal of Child Psychology and Psychiatry and Allied Disciplines* **41**, 645-655 (2000).

2 Rutter, M., Bailey, A. & Lord, C. *The social communication questionnaire: Manual*. (Western Psychological Services, 2003).

3 Nystrom, P., Gredeback, G., Bolte, S., Falck-Ytter, T. & EASE-team. Hypersensitive pupillary light reflex in infants at risk for autism. *Molecular Autism* **6**, 10 (2015).

4 Bergamin, O. & Kardon, R. H. Latency of the pupil light reflex: Sample rate, stimulus intensity, and variation in normal subjects. *Invest. Ophthalmol. Vis. Sci.* **44**, 1546-1554, doi:10.1167/iovs.02-0468 (2003).

5 Green, J. *et al.* Parent-mediated intervention versus no intervention for infants at high risk of autism: a parallel, single-blind, randomised trial. *Lancet Psychiatry*, doi:http://dx.doi.org/10.1016/S2215-0366(14)00091-1 (2015).

6 Daluwatte, C. *et al.* Atypical Pupillary Light Reflex and Heart Rate Variability in Children with Autism Spectrum Disorder. *Journal Of Autism And Developmental Disorders* **43**, 1910-1925, doi:10.1007/s10803-012-1741-3 (2013).
